# Dataset of a vanadium redox flow battery 10 membrane-electrode assembly stack

**DOI:** 10.1016/j.dib.2020.105840

**Published:** 2020-06-07

**Authors:** Artem T. Glazkov, Anatoly E. Antipov, Dmitry V. Konev, Roman D. Pichugov, Mikhail M. Petrov, Natalya V. Kartashova, Pavel A. Loktionov, Julia M. Averina, Ivan I. Plotko

**Affiliations:** a Mendeleev Russian University of Chemical Technology of Russia, Moscow, Russia; b Institute of Problems of Chemical Physics, Russian Academy of Sciences, Chernogolovka, Russia

**Keywords:** Redox flow battery, stack, energy storage, vanadium, cyclic voltammetry, capacity utilization, efficiency, flow cell

## Abstract

This paper contains a vanadium redox flow battery stack with an electrode surface area 40 cm^2^ test data. The aim of the study was to characterize the performance of the stack of the original design. The dataset include three series of galvanostatic charge-discharge cycling in the potential region 8–16 V with current densities 75, 150 and 200 mA/cm^2^ for 100 cycles. Coulomb, voltaic, energy efficiencies and capacity utilization coefficient are also provided for all three series.

Specifications Table

**Table:** 

Subject	Energy Engineering and Power Technology
Specific subject area	Design and testing of redox flow batteries
Type of data	Figure
How data were acquired	Potentiostat/galvanostat P-150X (ELECTRO CHEMICAL INSTRUMENTS), Mathcad, OriginPro
Data format	Raw, analyzed
Parameters for data collection	A laboratory prototype of the RFB stack was connected to a potentiostat/galvanostat by terminals at the ends of the stack and tested with the vanadium electrolyte of the following composition: 1 M VOSO_4_ in 4 M H_2_SO_4_ The potentiostat/galvanostat Elins P50Х (Russia) was connected to the PC to apply the ES8^Ⓡ^ software charge/discharge procedures.
Description of data collection	Synchronous registration of electrochemical data (current & potential), plot points registration speed: from one point per two seconds to one point per second.
Data source location	Mendeleev Russian University of Chemical Technology of Russia/ Moscow/Russia
Data accessibility	With the article

**Value of the Data**

• The data presented is a set of experiments to confirm the high electrochemical characteristics of a laboratory scale vanadium redox flow battery (VRFB) stack. It can be used to design the membrane electrode assemblies (MEA) on the industrial scale using the proposed materials and construction principles.

Scientists and researchers in the field of redox flow batteries development can benefit from the data provided. Moreover, one can use this dataset to verify the mathematical models of different RFB operating concepts under study.
The data presents a reliable basis for further scaling of the VRFB energy source via both the MEA area increase and the increase of the total number of MEAs in the stack
The additional value of the survey provided leans on the fact that this experiment proposes, to the best of authors knowledge, the first VRFB stack construction made in Russian Federation. The stack electrochemical measurements during electrolyte testing were carried out for the three values of the current applied to the stack. Based on this data one can predict the optimal modes of VRFB operation.

## 1 Data description

**Fig. 1:**
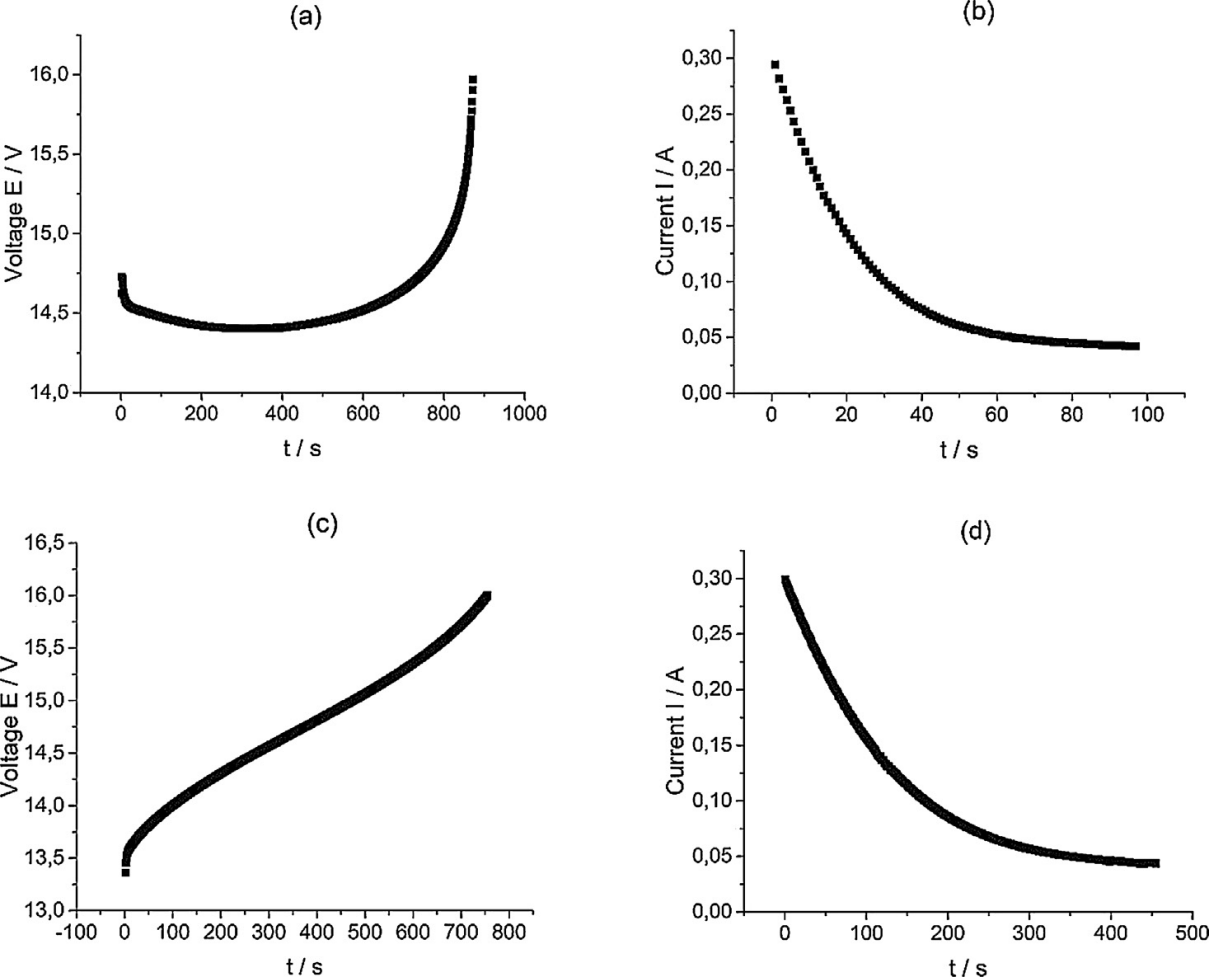
The process of the electrolyte preparation (initial composition 1 M VOSO_4_ in 4 M H_2_SO_4_, (2 × 51.6 ml) by alternating galvanostatic (a, c) and potentiostatic (b, d) electrolysis modes of a 10 MEA stack to reach the oxidation states of V^2+^ and *V* ^+^ ^5^ for the experiment with 300 mA current applied.

The dataset describes a series of electrochemical measurements on the VRFB [1], [2], [3] stack obtained with different values of the current imposed on the stack. Data is presented in the following composition:

1) Fig. 1 presents data of the electrolyte in the initial composition of 1 M VOSO_4_ in 4 M H_2_SO_4_ preparation process before the cyclic charge-discharge test of the 10 MEA stack under the 300 mA current applied conditions. The dataset (Fig.1) is provided in the Supplementary material «Raw data for Fig.1 a V-s, b A-s, c V-s, d A-s».

**Fig. 2:**
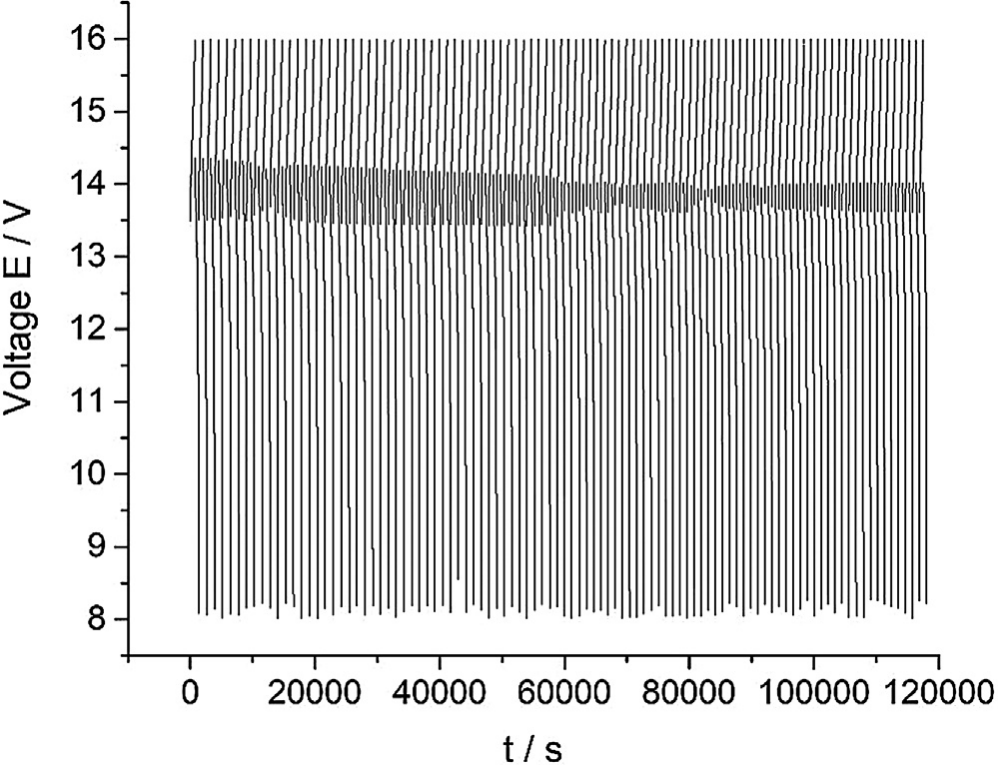
The data of the 10 MEA stack cyclic charge-discharge test under alternating galvanostatic mode with the parameters: magnitude of the applied current – 300 mA, current density – 75 mA/cm^2^, pumping speed 200 ml/s. The tests were performed at room temperature.

**Fig. 3:**
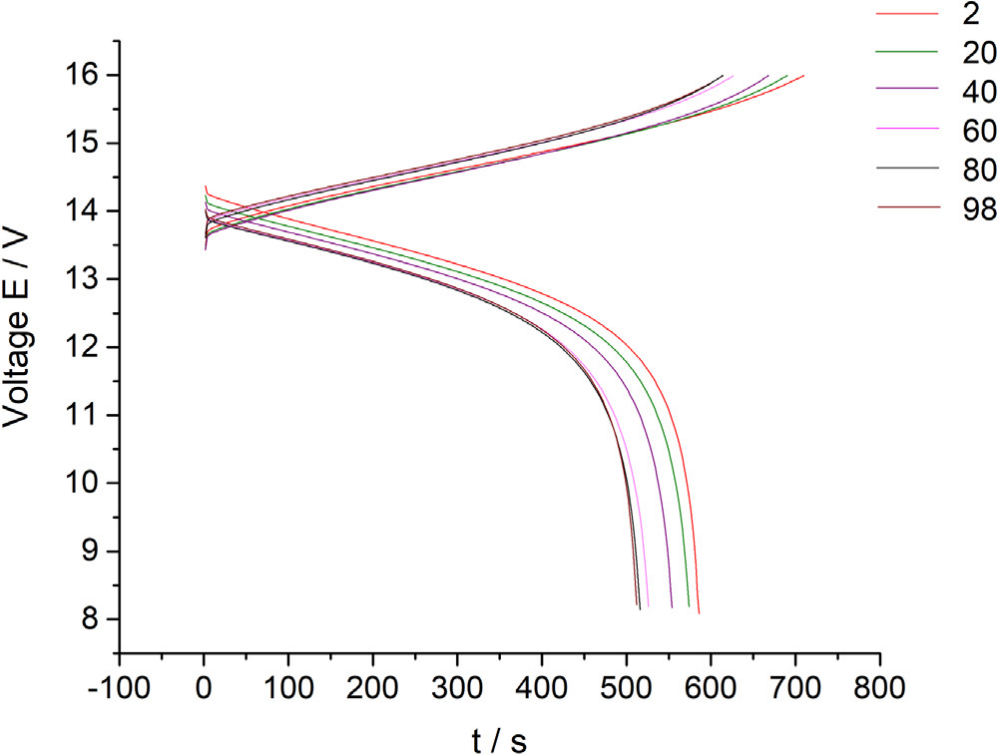
The dataset of the 10 MEA stack cyclic charge-discharge test in alternating galvanostatic mode with the parameters: magnitude of the applied current – 300 mA, current density – 75 mA/cm^2^, flow rate 200 ml/s. The tests were performed at room temperature (selected data for clarity purposes – every twentieth cycle of the 100 cycles experiment).

Fig. 2 presents data of a cyclic charge-discharge test of a 10 MEA stack while applying 300 mA current for 100 cycles (all data). The dataset (Fig.2) is provided in the Supplementary material «Raw data for Fig.2 and 3 V-s».

Fig. 3 presents data of the 10 MEA stack cyclic charge-discharge test under the 300 mA current applied (every twentieth cycle of the 100 cycles experiment presented on the Fig. 3 for clarity purposes). The dataset (Fig.3) is provided in the Supplementary material «Raw data for Fig.2 and 3 V-s».

**Fig. 4:**
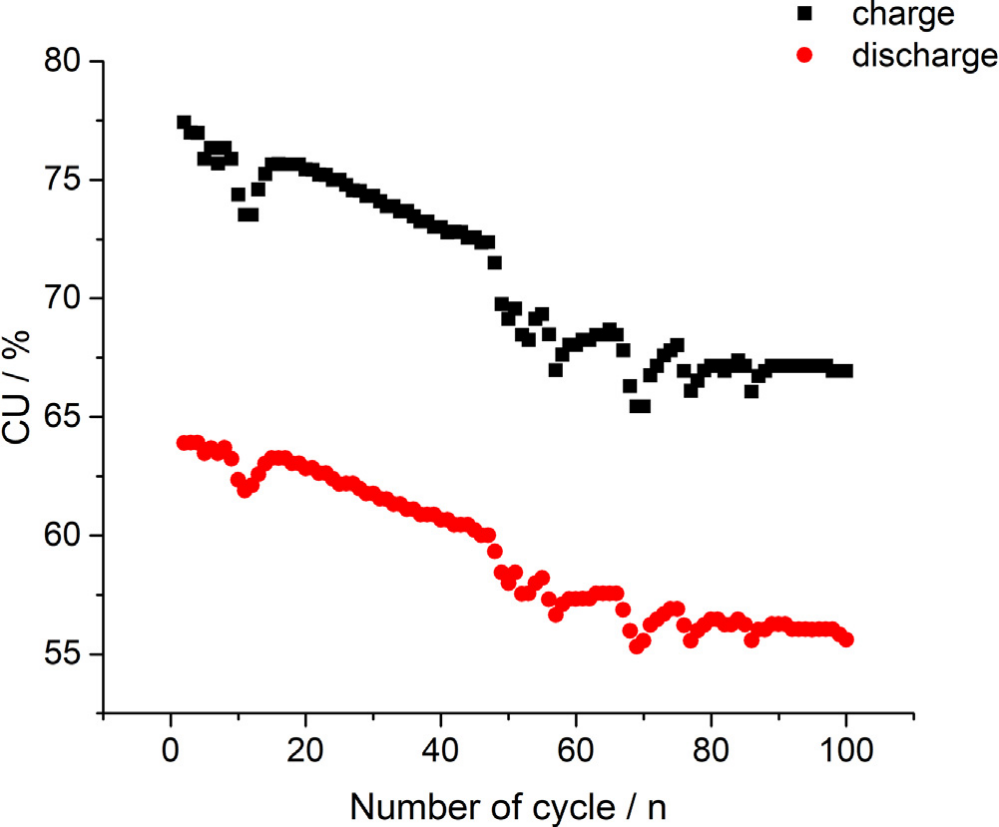
Calculation values of capacity utilization (CU) based on the data in Fig. 2 while 300 mA current being applied on the stack.

Fig. 4 presents capacity utilization (CU) calculation values based on the data in Fig. 2. The dataset (Fig.4) is provided in the Supplementary material «Raw data for Fig.4%-n». CU value (Figs. 4, 8 and 11) represents the ratio of the real amount of charge consumed/generated by stack at N(th) cycle of charge/discharge procedure to the theoretical charge needed for complete redox transformation of vanadium salts in electrolyte tanks.

**Fig. 5:**
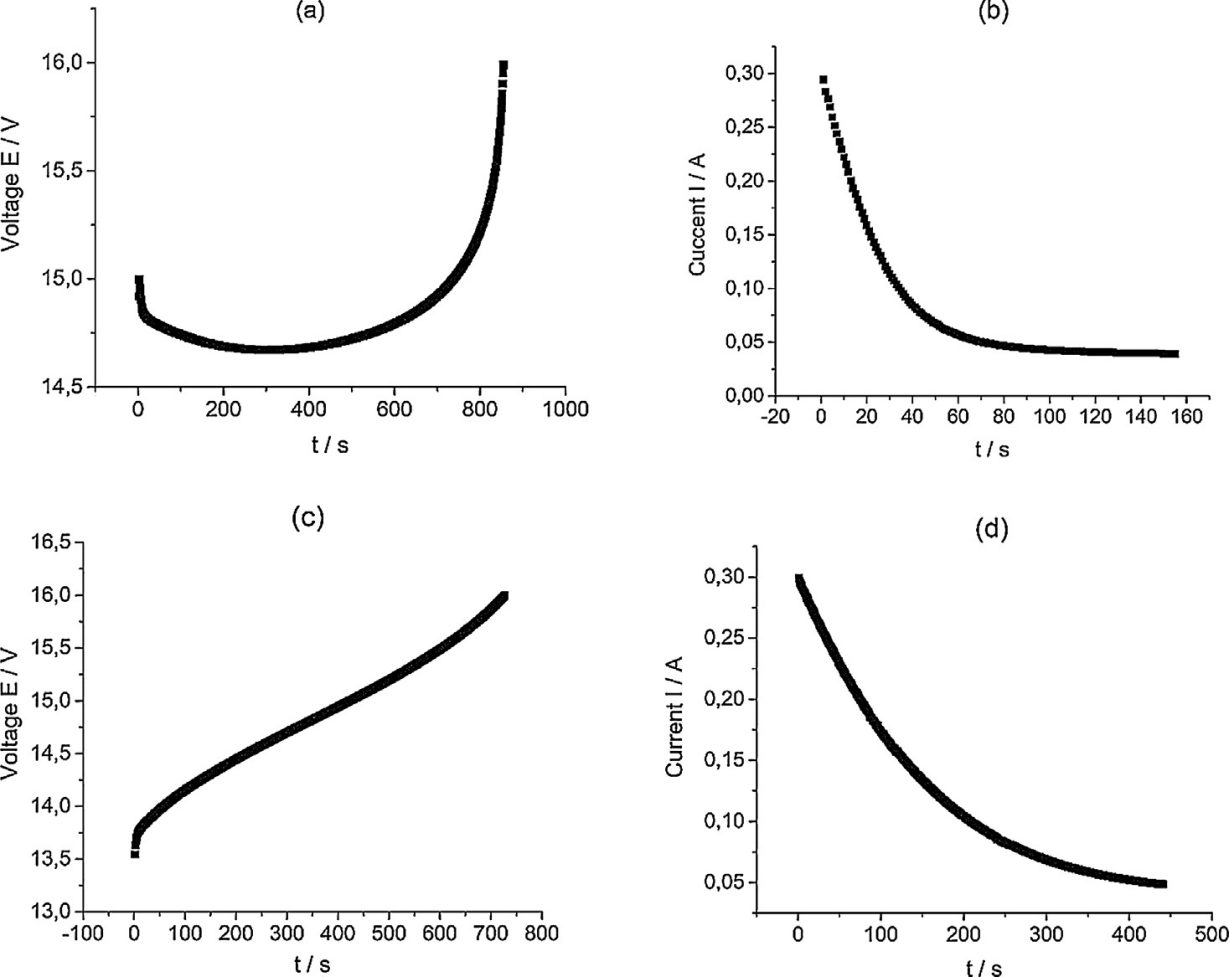
The process of the electrolyte preparation (initial composition 1 M VOSO_4_ in 4 M H_2_SO_4_, (2 × 51.6 ml) by alternating galvanostatic (a, c) and potentiostatic (b, d) electrolysis modes of a 10 MEA stack to reach the oxidation states of V^2+^ and *V* ^+^ ^5^ for the experiment with 600 and 800 mA currents applied.

1) Fig. 5 presents data of the electrolyte preparation process (the initial composition 1 M VOSO_4_ in 4 M H_2_SO_4_) to perform the 10 MEA stack cyclic charge-discharge test with 600 and 800 mA currents applied. The above graphs indicate a reproducible electrolyte charging process. The dataset (Fig.5) is provided in the Supplementary material «Raw data for Fig.5 a V-s, b A-s, c V-s, d A-s».

**Fig. 6:**
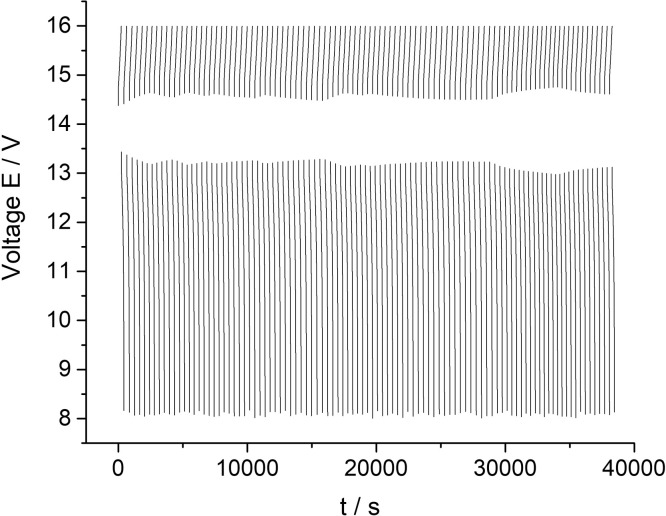
The dataset of the 10 MEA stack cyclic charge-discharge test in alternating galvanostatic mode with the parameters: magnitude of the applied current – 600 mA, current density – 150 mA/cm^2^, pumping speed 200 ml/min.

**Fig. 7:**
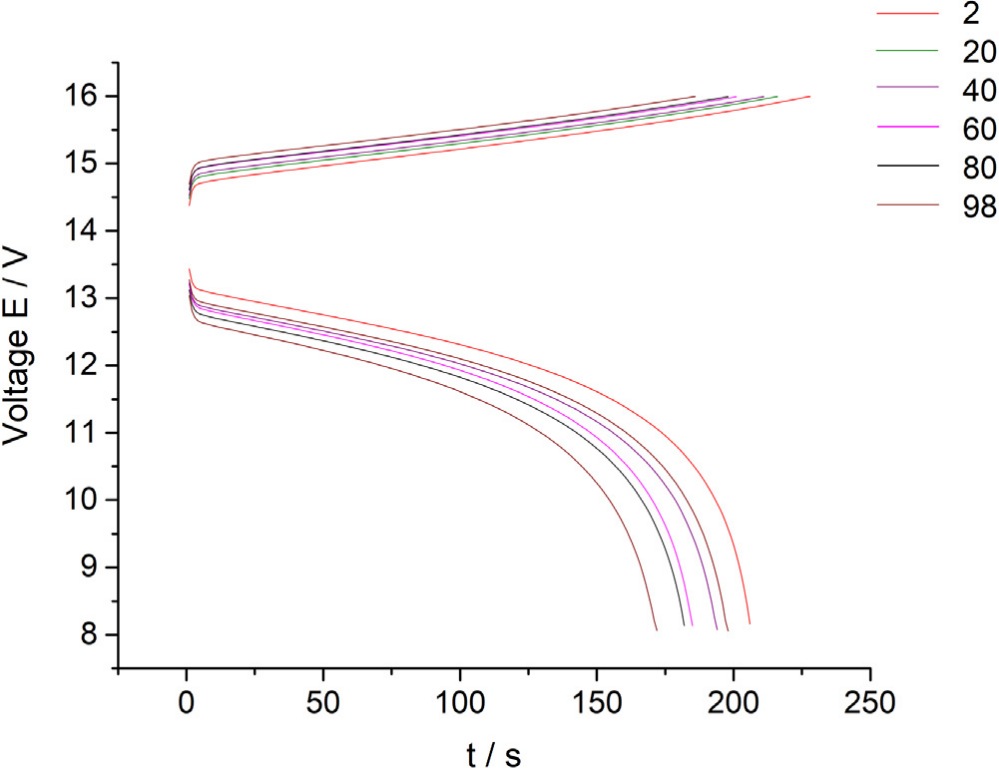
The dataset of the 10 MEA stack cyclic charge-discharge test in alternating galvanostatic mode with the parameters: magnitude of the applied current – 600 mA, current density – 150 mA/cm^2^, pumping speed 200 ml/s. The tests were carried out at room temperature (selected data for clarity purposes – every twentieth cycle of the 100 cycles experiment). The tests were at room temperature.

Fig. 6 presents data of 10 MEA stack cyclic charge-discharge test while applying 600 mA current for 100 cycles (all data). The dataset (Fig.6) is provided in the Supplementary material «Raw data for Fig.6 and 7 V-s».

Fig. 7 presents data of the 10 MEA stack cyclic charge-discharge test under the 600 mA current applied (every twentieth cycle of the 100 cycles experiment presented on the Fig. 6 for clarity purposes). The dataset (Fig.7) is provided in the Supplementary material «Raw data for Fig.6 and 7 V-s».

**Fig. 8:**
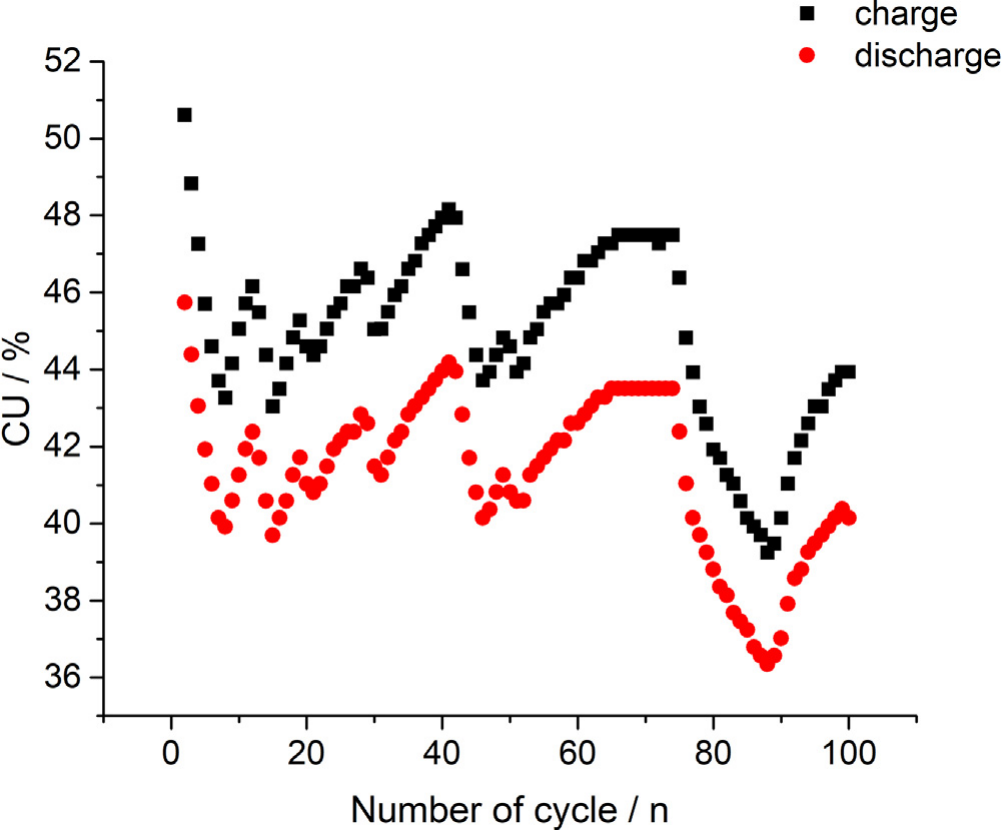
Calculation values of capacity utilization (CU) based on the data in Fig. 6 while 600 mA current being applied on the stack.

Fig. 8 presents capacity utilization (CU) calculation values based on the data in Fig. 6. The dataset (Fig.8) is provided in the Supplementary material «Raw data for Fig.8%-n».

**Fig. 9:**
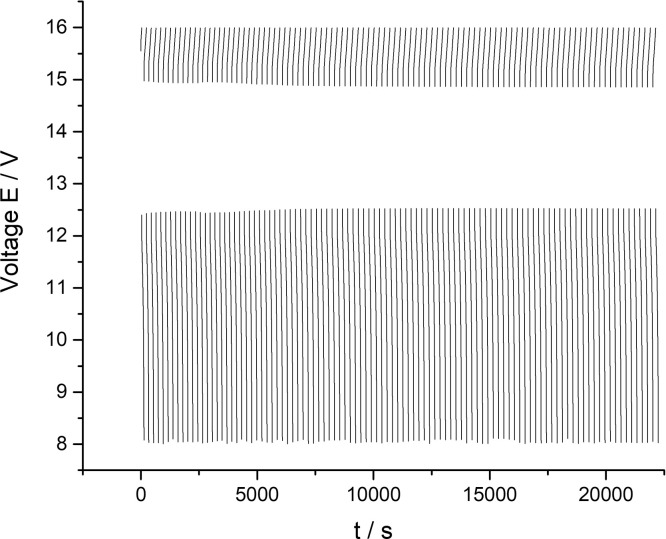
The dataset of the 10 MEA stack cyclic charge-discharge test under alternating galvanostatic mode with the parameters: magnitude of the applied current – 800 mA, current density – 200 mA/cm^2^, pumping speed 200 ml/min. The tests were performed at room temperature.

**Fig. 10:**
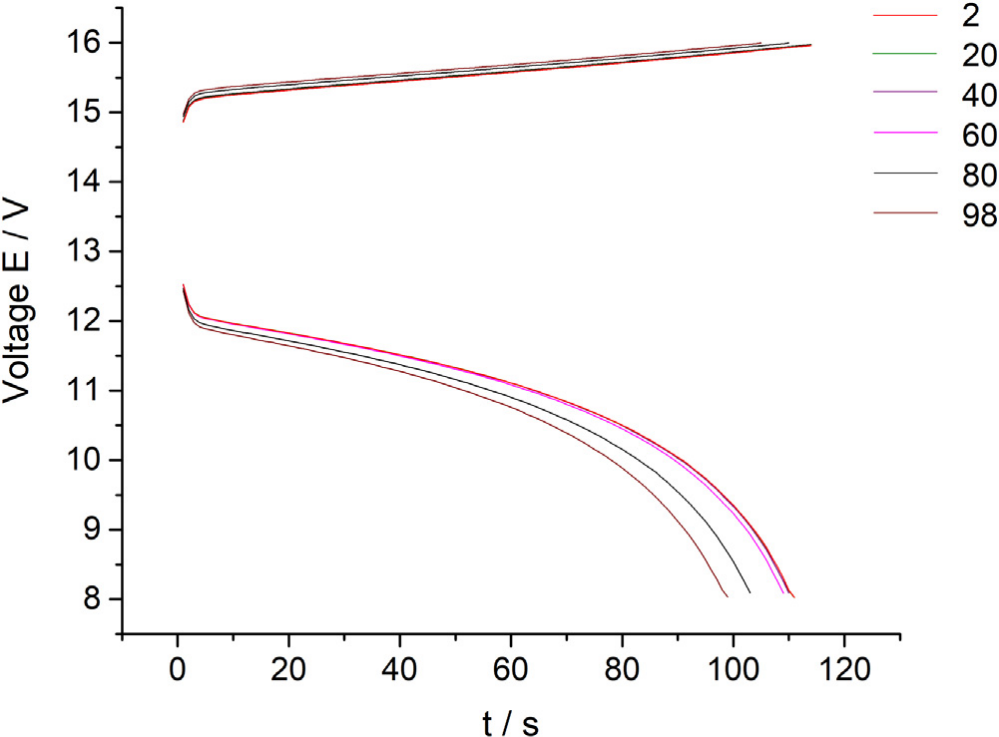
The dataset of the 10 MEA stack cyclic charge-discharge test in alternating galvanostatic mode with the parameters: magnitude of the applied current – 800 mA, current density – 200 mA/cm^2^, pumping speed 200 ml/s. The tests were performed at room temperature (selected data for clarity purposes – every twentieth cycle of the 100 cycles experiment).

Fig. 9 presents data of the 10 MEA stack cyclic charge-discharge test under 800 mA current applied for 100 cycles (all data). The dataset (Fig.9) is provided in the Supplementary material «Raw data for Fig.9 and 10 V-s».

Fig. 10 presents data of the 10 MEA stack cyclic charge-discharge test under the 800 mA current applied (every twentieth cycle of the 100 cycles experiment presented on the Fig. 9 for clarity purposes). The dataset (Fig.10) is provided in the Supplementary material «Raw data for Fig.9 and 10 V-s».

**Fig. 11:**
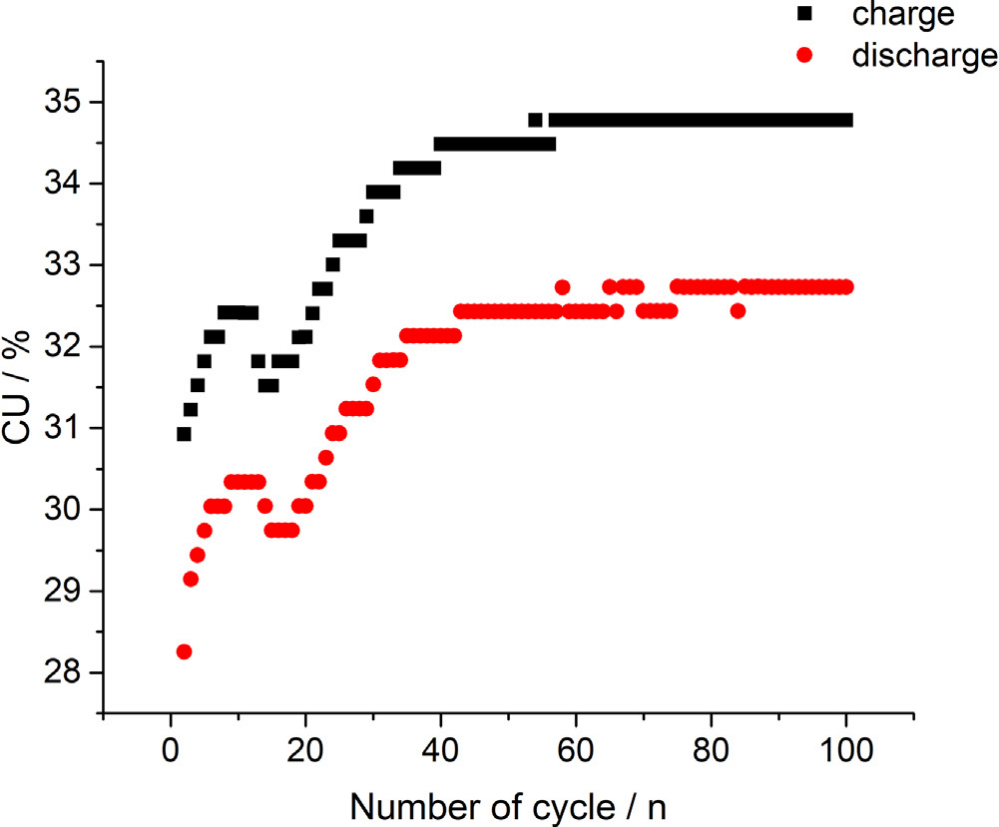
Calculation values of capacity utilization (CU) based on the data in Fig. 9 while 800 mA current being applied on the stack.

Fig. 11 presents capacity utilization (CU) calculation values based on the data in Fig. 9. The dataset (Fig.11) is provided in the Supplementary material «Raw data for Fig.11%-n».

**Fig. 12:**
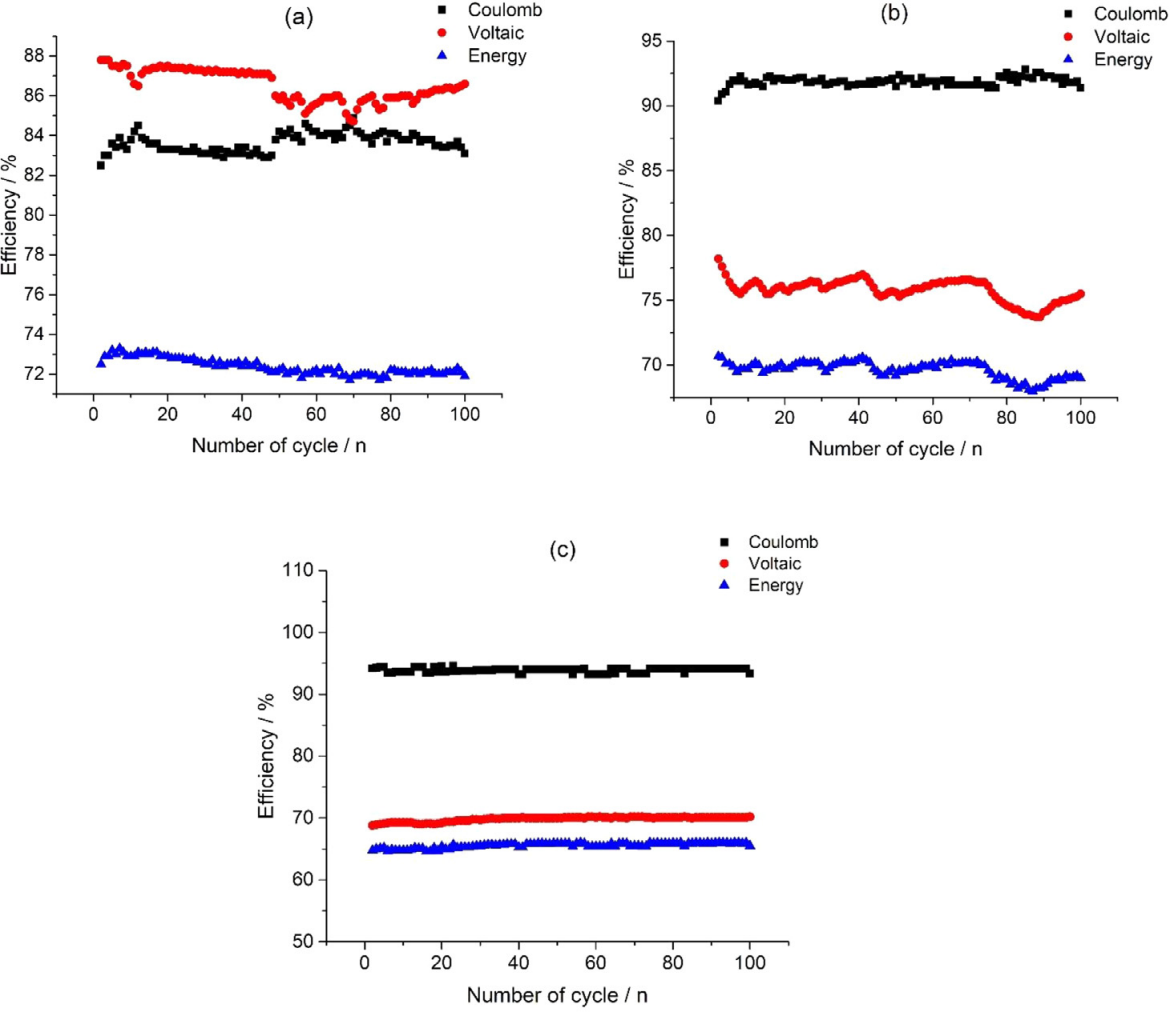
Data of a CE, VE and EE calculation for a different applied currents on the stack: (a) 300 mA, (b) 600 mA and (c) 800 mA.

Fig. 12 presents data of coulombic efficiency (CE), voltaic efficiency (VE) and energy efficiency (EE) calculation based on the data: in Fig. 2 for the 300 mA current, in Fig. 6 for the 600 mA current and in Fig. 9 for the 800 mA current, applied on the stack. The dataset (Fig.12) is provided in the Supplementary material «Raw data for Fig.12a%-n, Raw data for Fig.12b%-n and Raw data for Fig.12c%-n».

## 2 Experimental design, materials, and methods

The experimental setup included two reservoirs with an electrolyte of 1 M VOSO_4_ in 4 M H_2_SO_4_ composition, each connected to the input and output of the positive and negative electrode areas of the 10 MEА stack by tubes with an internal cross section of 3 mm. A peristaltic pump of variable capacity in the range (from 190 to 210 ml / min) was used in each circuit. For all electrochemical measurements potentiostat P-150 X (Elins, Russia) was used. Measurement procedures were specified using a PC in the ES8^Ⓡ^ software.

The stack represented a battery of 10 МЕА, each MEA consisted of 4 electrodes (material Sigraсet 39AA) with 2 cm^2^ area located on both sides of the LNGPF IEM 103 membrane. Current collector sheets as a part of bipolar plates were made of Graflex (carbon sheet material), treated according to a special procedure of impregnation with a fluoropolymer solution (application for an invention is filed "Modifying method of flexible graphite sheets used as slip rings for redox flow batteries" RU 2019111497U, 17.04.2019). As a flow field, 1 mm thick Teflon (fluoropolymer) was used with electrolyte distribution channels made by laser engraving. The stack was assembled between titanium end plates using PEEK fittings with internal thread. The assembly order and design of elements will be published shortly in a utility model patent (application for a utility model is filed "Device for a battery of membrane-electrode blocks of redox flow battery" RU 2020107750U, 20.02.2020).

The primary charging of 1 M VOSO_4_ in 4 M H_2_SO_4_ electrolyte was performed in two stages. At the first stage, a current value of 300 mA was set and held until a voltage of 16 V was reached. At the second stage, in a constant voltage mode of 16 V in magnitude, a current was recorded until its value decreased below 60 mA. Next, the contents of the tank with electrolytically electrolyte, converted from VOSO_4_ to *V* ^+^ ^5^, was replaced with a new portion of the electrolyte of the 1 M VOSO_4_ original composition and the two-step procedure was repeated.

During the charge–discharge cycling procedure, the stack was continuously fed with electrolytes at a constant rate (190–210 ml / min). The charge–discharge test procedure included alternating application of the 300, 600, or 800 mA constant current on the stack. The current direction was reversed when the voltage exceeded the following limits: the 16 V upper limit, the 8 V lower limit.

This regime corresponded to the similar single MEA testing conditions in the potential range from 1.6 to 0.8 V (the range can be decreased to reduce the amount of time to perform the experiment with the large number of charge–discharge cycles), which is widely used in studies of VRFB [4,5].

## CRediT authorship contribution statement

**Artem T. Glazkov:** Writing - original draft, Formal analysis. **Anatoly E. Antipov:** Writing - review & editing, Project administration. **Dmitry V. Konev:** Conceptualization. **Roman D. Pichugov:** Methodology. **Mikhail M. Petrov:** Resources. **Natalya V. Kartashova:** Data curation. **Pavel A. Loktionov:** Validation. **Julia M. Averina:** Visualization. **Ivan I. Plotko:** Software.

## Declaration of Competing Interest

The authors declare that they have no known competing financial interests or personal relationships which have, or could be perceived to have, influenced the work reported in this article.

## Appendix Supplementary materials

Image, application 1

Image, application 2

Image, application 3

Image, application 4

Image, application 5

Image, application 6

Image, application 7

Image, application 8

Image, application 9

Image, application 10

Image, application 11

Image, application 12
